# Feature-based attention warps the perception of visual features

**DOI:** 10.1038/s41598-023-33488-2

**Published:** 2023-04-20

**Authors:** Angus F. Chapman, Chaipat Chunharas, Viola S. Störmer

**Affiliations:** 1 Department of Psychology, UC San Diego, La Jolla, CA 92092 USA; 2 Cognitive Clinical and Computational Neuroscience Lab, KCMH Chula Neuroscience Center, Thai Red Cross Society, Department of Internal Medicine, Chulalongkorn University, Bangkok, 10330 Thailand; 3 Department of Brain and Psychological Sciences, Dartmouth College, Hanover, NH USA; 4 Department of Psychological and Brain Sciences, Boston University, 64 Cummington Mall, Boston, MA 02215 USA

**Keywords:** Human behaviour, Attention

## Abstract

Selective attention improves sensory processing of relevant information but can also impact the quality of perception. For example, attention increases visual discrimination performance and at the same time boosts apparent stimulus contrast of attended relative to unattended stimuli. Can attention also lead to perceptual distortions of visual representations? Optimal tuning accounts of attention suggest that processing is biased towards “off-tuned” features to maximize the signal-to-noise ratio in favor of the target, especially when targets and distractors are confusable. Here, we tested whether such tuning gives rise to phenomenological changes of visual features. We instructed participants to select a color among other colors in a visual search display and subsequently asked them to judge the appearance of the target color in a 2-alternative forced choice task. Participants consistently judged the target color to appear more dissimilar from the distractor color in feature space. Critically, the magnitude of these perceptual biases varied systematically with the similarity between target and distractor colors during search, indicating that attentional tuning quickly adapts to current task demands. In control experiments we rule out possible non-attentional explanations such as color contrast or memory effects. Overall, our results demonstrate that selective attention warps the representational geometry of color space, resulting in profound perceptual changes across large swaths of feature space. Broadly, these results indicate that efficient attentional selection can come at a perceptual cost by distorting our sensory experience.

## Introduction

Our perceptual experience is strongly influenced by attention. At any given moment, our senses are confronted with an abundant amount of information, exceeding our mind’s capacity to process that information effectively. Selective attention enables us to prioritize relevant information over irrelevant information. Attention can be allocated to specific regions in the visual field or to visual features, such as a color or orientation, and in both cases results in increased performance for attended relative to unattended information^1–4^. These behavioral improvements are accompanied by increases in the activity of neural populations associated with the attended spatial location^5–9^ or visual feature^10–13^, consistent with theories proposing that attention increases the gain of neurons most selective for the relevant stimulus^1,11^.

More recent evidence indicates that selective attention not only improves sensory processing, but can also have profound effects on our subjective visual experience, that is, how an attended visual stimulus appears to the observer. Several studies have demonstrated that spatially attending to a visual object increases the apparent intensity of that stimulus, making it appear higher contrast or more saturated than an unattended stimulus^14–18^, in line with neurophysiological findings showing that attention alters the strength of a stimulus by increasing its effective contrast^7,19–21^. In these cases, gain modulation models can explain the changes in both perception and performance related to attended stimuli.

Critically, some theories of feature-based attention propose selection can occur in ways other than via gain modulation of target-tuned neurons, especially in complex visual scenes where distractor features may be easily confusable with the target feature. Optimal tuning accounts, as they are commonly referred to, instead propose that when targets and distractors are similar, attention may enhance neurons that are tuned away from the target feature to maximize the relative saliency of the target stimulus^22–24^. Specifically, when selecting a target among similar distractors (e.g., an orange item among yellow items), increasing the signal of an exaggerated target feature that is less similar to the distractor (e.g., red–orange) would provide a higher signal-to-noise ratio relative to selecting the exact target feature. While previous studies have found that human observers enhance processing of the most informative feature value given the experimental context, not necessarily the target feature itself^23,24^, it is unknown how such enhancements would impact the perceptual experience of target and nontarget features. We predicted that if attention-induced modulations of neural responses are linked to changes in appearance, then selection would distort feature representations in systematic ways such that the perceived target would be biased away from the distractor feature. Indeed, the current study demonstrates profound distortions in color perception due to attention: if an orange item is selected among yellow distractors, the selected target color appears more red-like than it actually is. Importantly, we also show that these distortions affect large swaths of feature space, indicating that attentional enhancement to an off-tuned color in one part of the feature space alters the underlying representational geometry globally. We are the first to show such dramatic warping in representational spaces due to attention, discovering that our subjective experience of the world is often distorted to optimize information processing.

## Results

### Experiment 1: perception of an attended color is biased away from distractor colors

**Figure 1:**
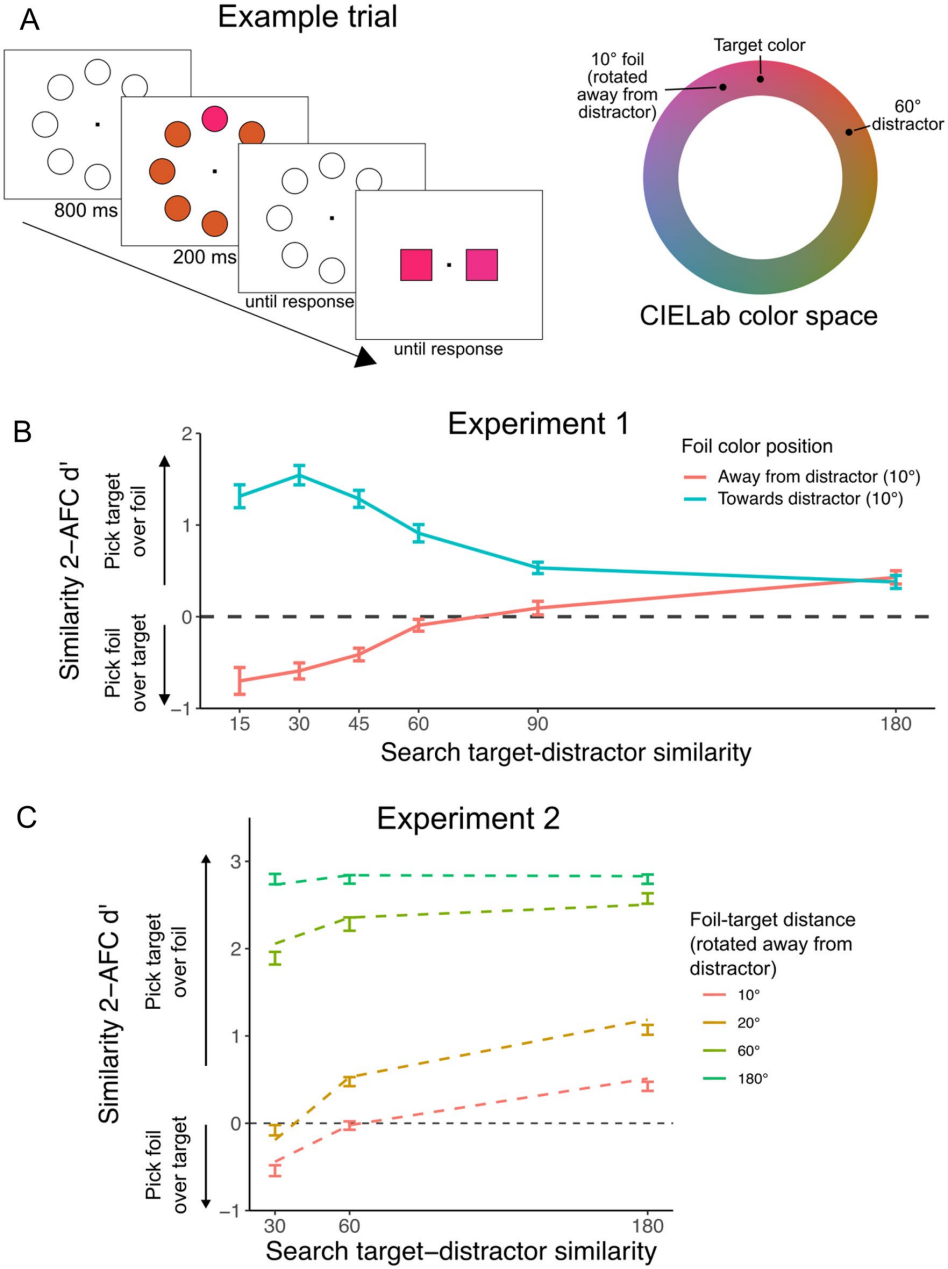
(**A**) Structure of an example experiment trial. Participants first searched for an oddball target item among distractors. Following report of the target’s location, they then reported which of two colors was most similar to the previous visual search target. All colors were selected from a 360° CIELab color space, and we systematically manipulated target-distractor similarity during visual search as well as the target-foil distance during the 2-AFC. Here an example is shown with a 60° color distance between target and distractor during visual search, and a foil color 10° different from the target, rotated in the direction away from the distractor color. (**B**) Results from Experiment 1. 2-AFC similarity judgment performance (d′) was significantly affected by the foil color and target-distractor similarity: Participants chose the target color more often when the foil was rotated towards the distractor (positive d′), but chose the foil color more often when it was rotated away for the distractor (negative d′) especially for similar target-distractor conditions. (**C**) Results from Experiment 2. 2-AFC similarity judgment performance increased with less similar visual search distractors and greater foil distances. Foils were always rotated away from the distractor in this experiment. Dashed lines show fits from a signal detection model that allowed for variations in bias and strength of the target representation. Best fitting models suggested that 30° and 60° distractors resulted in a bias of 11.5° and 5.3°, respectively.

We investigated how feature-based attention impacts the perception of target features across a series of experiments. To engage feature-based attention, we used a simple visual search task in which participants were presented with an array of briefly (200 ms) presented colored circles to locate an oddball target among distractors (see Fig. 1A). The target color was chosen randomly on each trial from a 360° CIELab color space, while the distractor was chosen at specific feature distances from this target to be more or less perceptually similar. Critically, the target and distractor colors changed at random from trial to trial, and participants were not cued in advance about the target nor distractor colors; thus, this set-up removed the possibility for participants to know something about the target or distractor color in advance and potentially bias their attention in anticipation of the upcoming features. Instead, finding the oddball during search induced feature-based attention. To probe how attentional selection would modulate the perception of the target color, immediately after reporting the target item’s location, participants performed a 2-alternative forced choice (2AFC) task reporting which of two squares was most similar in color to the previously selected target item. Of the two squares, one was always presented in the exact target color, while the other was a foil color chosen to be 10° away from the target on the color wheel, either in the direction towards the search distractor, or away from it (i.e., more similar to the distractor or less similar, Fig. 1A). The logic of our experimental design was as follows: by selecting the target color amongst distractors during search, attention may bias the target representation to increase separability between target and distractor, and we can assess these biases in the secondary psychophysical judgment task. Thus, any observed biases in the 2AFC task are ultimately driven by selection during search itself.

The main question was whether participants’ color reports during the 2AFC were influenced by the relation between the distractor color during search and the foil color in the 2AFC. If selective attention is tuned away from the distractor color to more effectively select the target, as predicted by optimal tuning accounts, then the target color itself might appear shifted away from the distractor color. In this case, participants should choose the foil color more often than the target when it is more distant from the distractor color (i.e., rotated away from the distractor on the color wheel). Conversely, when the foil is less distant from the distractor (rotated towards the distractor), participants should choose the target color more often than the foil. Our results demonstrate that this was the case: Participants’ (n = 30) responses in the 2AFC were strongly influenced by the relative feature distance between the foil color of the 2AFC and the distractor color during search (F = 182.06, *P* < 0.001, $\upeta_{\text{p}}^2$ = 0.863). Participants chose the foil color more often than the target color when the foil was rotated away from the distractor, apparent as a negative d′﻿ in 2AFC performance; and they chose the correct target color more often when the foil color was rotated towards the search distractor color, evident as a positive (and quite large) d′﻿ in the 2AFC (Fig. 1B). This pattern implies that the appearance of the target color was distorted and shifted away from distractor colors. Importantly, the magnitude of this bias was affected by the similarity of targets and distractors during search (*F* = 58.37, *P* < 0.001, ﻿$\upeta_{\text{p}}^2$ = 0.668), with the largest bias present for trials with highly similar distractors (15°–45°, Fig. 1B). However, biases were significant for all target-distractor distances below 180° around the color wheel (*p*’s < 0.001). This suggests that feature-based attention alters color perception across a range of difficult search tasks, with particularly strong distortions when target-distractor similarity is high.

### Experiment 2: the magnitude of induced biases depends on target-distractor similarities

To estimate the magnitude of attention-induced perceptual biases, in Experiment 2, we manipulated the feature distance between the target and foil colors during the 2AFC similarity judgment as well as the similarity between targets and distractors during visual search (n = 50). To ensure a sufficient number of trials, in this experiment, all foils were rotated away from the visual search distractor on the color wheel at 10°, 20°, 60°, or 180° from the target. We found a highly significant interaction between foil distance during the 2AFC and target-distractor similarity during search (*F* = 23.6, *P* < 0.001, $\upeta_{\text{p}}^2$ = 0.325). When the foil color was rotated 10° away from the target color, we replicated the pattern of performance found in the first experiment, with participants selecting the foil more often when the visual search distractor was more similar to the target (30° away in color space, Fig. 1C). Increasing the distance between the target and foil colors resulted in better discrimination of the target color, however we found that smaller target-distractor similarities during visual search still affected performance when the foil was up to 60° from the target color (*p*’s < 0.001). These findings indicate that distractors that are similar to targets during visual search can have large biasing effects on the perception of colors across the feature space.

Notably, this experiment revealed two conditions in which similarity judgments, on average, were at near-chance performance (d´ = 0 for 60° target-distractor similarity during search, 10° foil distance; and 30° target-distractor similarity during search, 20° foil distance; Fig. 1C). Such conditions, where participants choose the target and foil color equally often, suggest that participants perceived the two choices to be equidistant from their in-mind target representations. Thus, using these conditions we can infer the magnitude of the bias induced by the distractor color for different target-distractor similarities: the results suggest that for high target-distractor similarities of 30°, the perceptual bias is approximately 10° in color space, while it is about half (5°) when target-distractor similarity is relatively low (60° on the color wheel). In support of this interpretation, we fit the full experimental data with a model based on signal detection theory (see SI Methods)^25^, and found that this model fit the data best when biases of about these magnitudes were included in the target representation for target-distractor distances of 30° (bias = 11.5°) and 60° (bias = 5.3°).

### Experiment 3: Biases are not driven by differences in temporal delay

**Figure 2:**
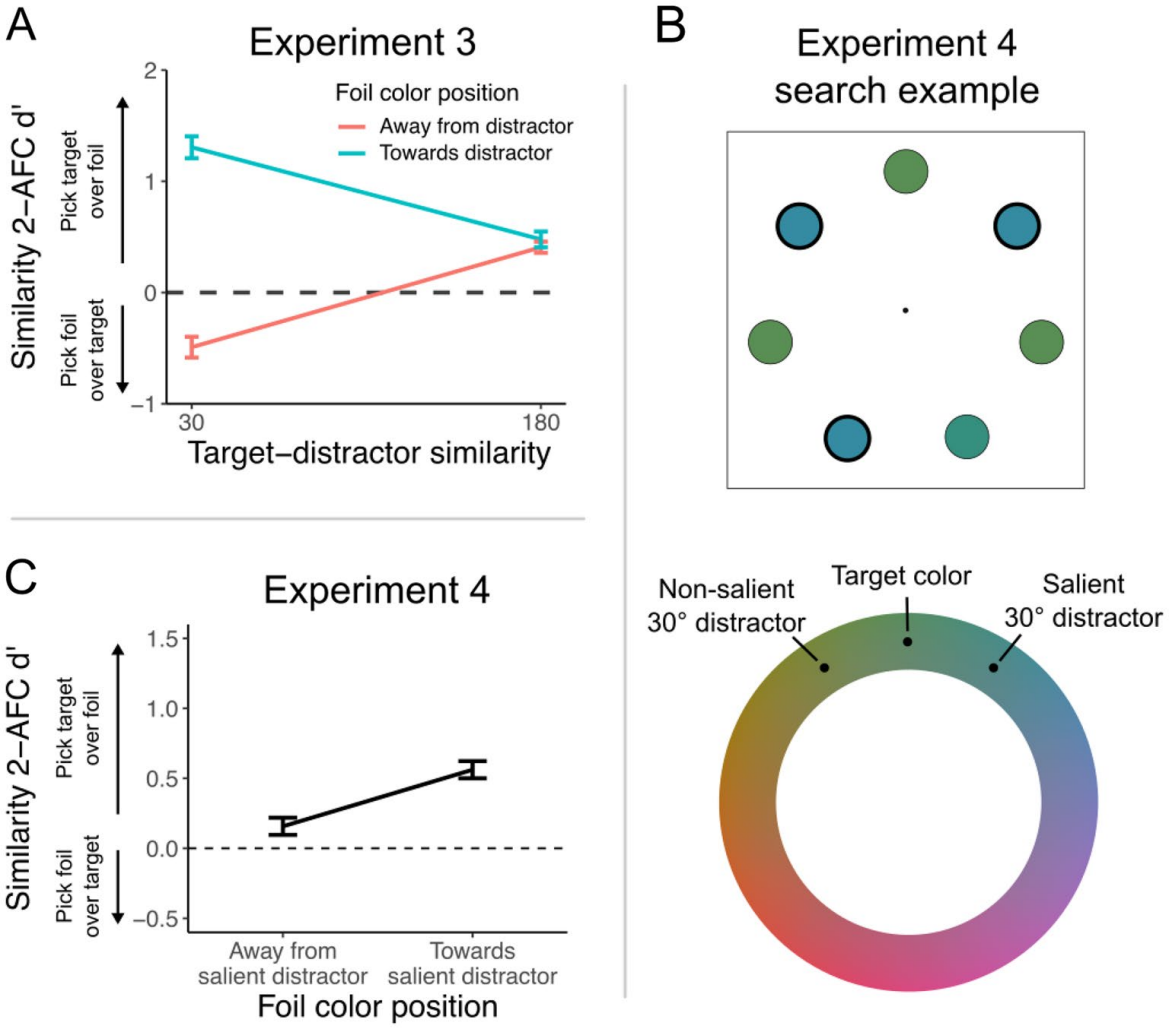
(**A**) Results from Experiment 3, where there was no delay between the search array and similarity judgment. Color judgment performance in the 2AFC task was comparable to Experiment 1 for 30° target-distractor similarity during search. (**B**) Example of a visual search trial in Experiment 4. Participants searched for an oddball target color (bottom right in this example) among distractors that were evenly chosen 30° clockwise and counterclockwise relative to the target. One distractor color was made more salient by increasing the thickness of its border. (**C**) Results from Experiment 4, where we manipulated the salience of distractors from one direction around the color wheel. Perception of the target color was more strongly biased by the salient distractor.

Delays between encoding and report can exaggerate biases in memory for visual features^26–28^, and although including RT as a covariate in a reanalysis of Experiment 1 data did not explain the perceptual biases we found (see SI Methods), we wanted to confirm this experimentally by fixing the delay across conditions. Thus, we ran an experiment in which participants (n = 30) completed a similar task, however following the presentation of the search array they either responded with the location of the target or immediately made a 2AFC similarity judgment. Despite the fact that there was no delay between the search presentation and the similarity response, and that the timing was now the same across all conditions, we still found a large bias for trials with target-distractor similarities of 30° (*t* = 13.75, *P* < 0.001, d = 2.51, Fig. 2A), while there was no bias with 180° similarity (*t* = 1.01, *P* = 0.321, d = 0.18; interaction: *F* = 102.61, *P* < 0.001, $\upeta_{\text{p}}^2$ = 0.780). Importantly, the magnitude of this bias did not differ when compared with the same condition in the first experiment (*F* = 2.52, *P* = 0.118). Thus, the perceived color distortions cannot be explained by longer RTs in some conditions than others, but they occur robustly even when there is no temporal delay between selection (visual search) and report (2AFC).

### Experiment 4: biases are induced by attention and not low-level visual properties

In each of the previous experiments, visual search arrays contained a single target surrounded by distractors in another color. Thus, low-level visual properties of these arrays, like those involved in simultaneous contrast effects^29^, rather than feature-based selection of the target itself, might influence the biases we have observed. To account for this possibility, we conducted a version of the experiment in which participants (n = 44) searched for an oddball target color amongst a set of distractors that were rotated 30° in each direction from the target (6 distractors total, 3 rotated clockwise, 3 counterclockwise, see Fig. 2B), negating any potential low-level perceptual effects. To assess the influence of attention on perceptual biases, on each trial distractors rotated in one direction were made more salient by increasing the size of the items’ border^30^. When similarity judgments were compared based on whether the foil was rotated towards the salient or non-salient distractor color, we found that the salient distractor induced a clear bias: participants chose the target more often when foils were rotated towards the salient distractor (*t* = 6.57, *P* < 0.001, d = 0.99, Fig. 2C). Therefore, attention drives the perceptual biases observed in these experiments, rather than low-level visual properties.

### Simulation: warping can be explained by changes in representational geometry

**Figure 3:**
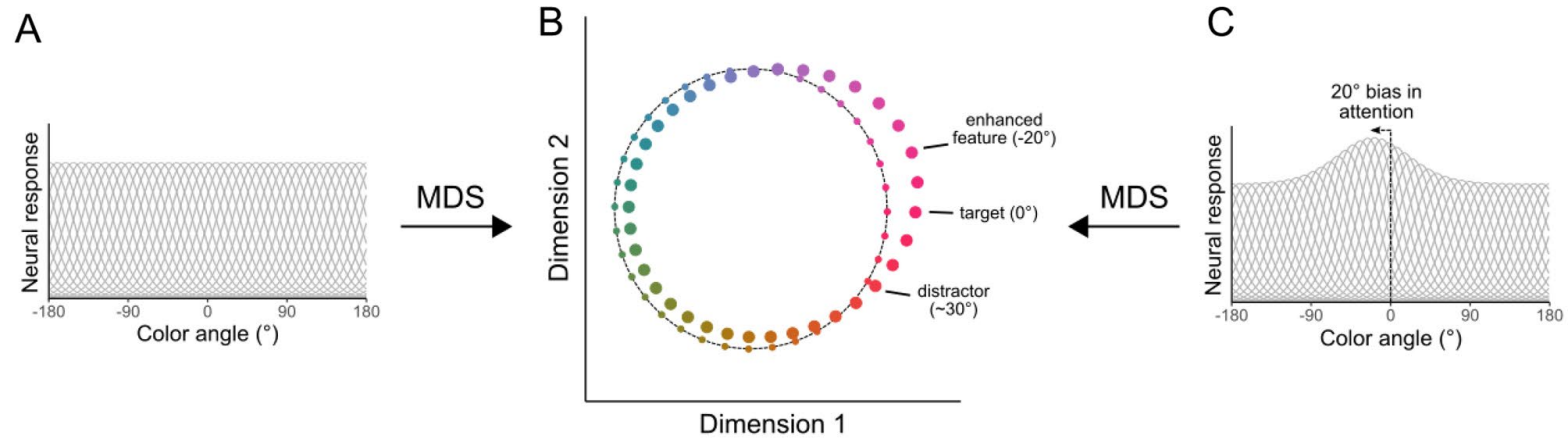
(**A**) Hypothetical neural population representing a circular feature space. (**B**) Multidimensional scaling (MDS) of the population results in a circular representational geometry (small circles overlayed on dashed line). Points in MDS space were subsampled to represent colors 10° in the feature space. (**C**) Neural population with simulated attentional gain applied at 20° from the true target position (0°), resulting in distortions in the representational geometry with expansion of the representation that is maximal around the center of the attentional gain (large circles in **B**).

How might the observed behavioral biases be accounted for at the level of neural representations? To explore this question, we took inspiration from the literature on representational geometry^31,32^. Representational geometry aims to understand how information about a stimulus is carried within neural activity, primarily through assessing the similarity or dissimilarity in activity patterns for different stimuli. To that end, we first simulated a population of neurons, with each neuron tuned to a different color within our circular feature space, and then performed multidimensional scaling (MDS) based on the Euclidian distance between each pair of hypothetical neurons. As expected, this MDS resulted in a circular geometry (Fig. 3A,B, small circles), demonstrating that the simulated population encoded the circular feature space. We then simulated the effects of attention by modulating the gain of each neuron based on its preference for a particular feature and repeating the MDS analysis, which resulted in a distortion of the feature space, expanding the representation near the target feature, and compressing the representation at the opposite end of the feature space (Fig. 3B, large circles).

This change in the representational geometry relates to our findings when considering gain applied to an off-tuned feature: for a given target and distractor, 30° apart in feature space, enhancement of neurons tuned away from the distractor (Fig. 3C) will result in an expansion of the representation centered on that off-tuned feature (Fig. 3B, large circles). Subsequently, this results in an asymmetry in the representational space, with the distance between the target and a 10° foil depending on the position of the foil relative to the distractor. In the representational space, there is a greater distance between the target and a foil rotated away from the distractor, consistent with the repulsion effects we find behaviorally. Thus, changes in the representational geometry of color space provide a novel explanation for the biases in perception driven by salient distractors.

## Discussion

It is well established that attention enhances our perceptual capacities by improving the detection or discrimination of relevant information in our environment. Here, we used visual search for an oddball target color to induce feature-based attention and assessed the perception of the target color in a subsequent similarity judgment task. Our findings show that feature-based attention can lead to quite dramatic distortions in perceptual processing, possibly by causing asymmetric shifts in representational geometry due to the enhancement of off-tuned neurons. Specifically, our results demonstrate that selecting an oddball color during visual search can distort the appearance of a target color away from a perceptually similar visual distractor, and that these distortions occur across large parts of the feature space. Interestingly, we observed these perceptual biases in a task where the target and distractor colors varied from one trial to the next and participants were not informed about what the target or distractor colors would be. Thus, feature-based attention as engaged by the oddball visual search task rapidly and reactively stretched and compressed different parts of the feature space to increase target-distractor dissimilarity, thereby supporting target selection effectively.

Broadly, the present results relate to other recent research that has shown attention-induced changes in perception, for example studies showing that attention increases the apparent contrast of attended relative to unattended stimuli^14,16,17,33,34^. These increases in apparent stimulus contrast are a natural consequence of gain modulation and help to strengthen target representations, thus aiding target detection and discrimination performance; at the same time, these changes render the stimulus representation non-veridical. Our results add to this literature on how attention can alter stimulus representations by showing that feature-based selection can induce changes in stimulus appearance that are non-orthogonal with respect to the relevant feature, resulting in less accurate feature representations. The extent to which attentional selection induces these distortions may in part depend on task demands: perceptual biases might be reduced in situations where veridical representations are more important, although potentially at the cost of less efficient attentional selection.

The perceptual biases observed in our study also have strong connections to previous models of (predominantly spatial) attention. Specifically, a number of attentional mechanisms have been identified that can account for the present effects. For example, computational models propose that feature-based attention (similar to spatial attention) operates via center-surround selection in feature space, allowing for isolation of the target from nearby distractor features^35,36^. These predictions have been supported by behavioral and electrophysiological findings that feature representations similar to the attended feature (i.e., nearby in feature space) are suppressed relative to more distant features^13,37–40^. Other studies suggest that feature tuning can be sharpened around the focus of attention^41,42^, or that additional neural populations are recruited to allocate additional resources towards the selected feature (i.e., receptive field recruitment)^43–46^. In all three cases, the result would be an overall skewed neural population response, effectively attenuating color representations nearby the selected color, which could cause perceptual distortions like those we observed here. However, since we found that perception was biased away from distractor colors, each of these proposed mechanisms would need to incorporate asymmetries (e.g., a shift in the “center” and/or stronger surround suppression of features in the direction of the distractor) to account for the specific biases we observe. Importantly all these changes in neural populations can lead to similar changes in representational geometry, as the same geometry can be observed from different underlying tuning properties^31^. Additionally, these mechanisms are similar to what has previously been proposed in spatial attention tasks that induced perceived position shifts of targets^47^, suggesting spatial and feature-based attention bias perception of inputs in comparable ways. Because visual cortex contains topographic representations of features such as color^48–50^ in addition to spatial retinotopic maps, the same proposed mechanisms could readily explain the findings of our experiments. Thus, similar to perceived location distortions in spatial attention, feature-based attention can distort the perception of features and warp feature-based representational maps, such as color.

A number of studies have shown that biases exist in working memory, such that items in memory can be attracted towards or repelled from each other^26–28,30^. These reported biases in memory are proposed to be the consequence of actively maintaining multiple representations at once, with some models demonstrating that representations naturally drift when held in memory for extended durations^51,52^. In contrast to these memory effects, our findings demonstrate that biases in perceived target color can occur even when only a single item is task-relevant, needs to be selected among another nontarget color in a briefly presented display, and is reported right after with only a minimal temporal delay (Exp. 3). This shows that perceptual representations are distorted at even the earliest stages of visual processing, before they enter into working memory and compete with other representations during storage. Additionally, recent work suggests that attentional templates of target colors can become biased by learning within a consistent distractor context^53^, suggesting that the perceptual effects we observed in our study may be consolidated in statistically more regular situations. In working memory studies, such biases have been attributed to adaptive distortions that improve memory for an overall set of items at the cost of accuracy (but not necessarily precision) for individual items^26^. A similar explanation can be made regarding our findings, as the bias increases the representational distance between target and distractor colors, effectively improving attentional efficiency. The cost of this improved efficiency is that perception of the target is warped from its veridical feature value. We hypothesize that warping happens as a byproduct of selection: representations of the target and distractor features are rapidly extracted from the visual search display; through recurrent activity, or at higher levels of representation, attention can intervene to delineate the different features and separate their representations to enable prioritization of the relevant information, and support efficient guidance to the target. Prioritization can mean on-target enhancement when features are distinct enough from one another, but when neural representations are more similar and overlapping, other mechanisms such as off-target enhancement can provide more efficient target selection. This means that the role of attention is not necessarily to directly enhance the relevant features, as is often assumed, but to shape representational spaces in a way that separates relevant from irrelevant information for more effective selection. Such warping may also serve to exaggerate and increase the detectability of changes in the environment^54^.

Importantly, our findings cannot be explained by basic perceptual effects, such as aftereffects or simultaneous contrast, which can also result in altered perception of visual features. Aftereffects typically take several seconds of stimulus presentation to emerge^55,56^, whereas our visual search arrays were presented for only 200–400 ms at a time. Although simultaneous contrast can occur much more quickly than aftereffects, it is typically observed when one color is fully surrounded by another^29,57^. Our color stimuli were physically separated on the display and delineated by borders^58^, meaning low-level interactions are likely negligible. Furthermore, we showed that with a balanced stimulus display, where the contrast between the target and distractors was effectively “cancelled out” (Exp. 4), perception was still biased by whichever distractors were most salient, demonstrating that attention is the primary driving factor behind the warping effects we observed.

In sum, our study provides strong evidence that attention warps representational spaces of visual features, resulting in biased perception of target features. Further, our simulation shows that these perceptual changes can be explained by changes in the underlying representational geometry. Such findings support models of attention that emphasize the relationship between target and distractor features^59^, such as optimal tuning accounts^22,23^, and argue against those that focus primarily on the target feature itself. Along with comparable findings in spatial attention^47^, this suggests that warping may be a general principle of attention, with perceptual representations undergoing rapid and adaptive changes when attention acts to separate items that are similar.

## Methods

### Participants

All experiments were conducted online, with participants recruited from the University of California, San Diego undergraduate subject pool and compensated with course credit. Participants gave informed consent before starting the experiment. All experiment procedures were carried out in accordance with standard guidelines and all protocols were approved by the Institutional Review Board at UC San Diego. Experiment 1 consisted of 30 participants (25 women, 5 men; age 20.4 ± 1.6 years). Experiment 2 consisted of 50 participants (42 women, 5 men, 3 did not report their gender; age 20.6 ± 1.7 years), with an additional 2 participants who were excluded with < 70% accuracy in visual search. Experiment 3 consisted of 30 participants (20 women, 10 men; age 20.3 ± 2.6 years). Experiment 4 consisted of 50 participants (33 women, 7 men, 4 did not report their gender; age 20.5 ± 2.1 years), with an additional 9 participants excluded with < 40% accuracy during visual search (this exclusion criteria was lower than in the other experiments because the more difficult task resulted in lower overall accuracy).

### Stimuli

Participants completed the experiments online on their own personal computer. Stimulus sizes are given in pixels, given that we had limited control over the display size and viewing distance. However, the display size was restricted to a minimum of 800 × 600 pixels, and participants were instructed to complete the experiment in full screen.

All colors were selected from a set of 360 equally spaced colors in a fixed-luminance plane of CIELab color space, drawn from a circle with radius 49 units, centered at L = 54, a = 21.5, b = 11.5. The visual search array consisted of 8 circles (80px diameter) arranged evenly in a ring centered on fixation (10px diameter filled black dot), except for Experiment 4 in which there were 7 circles evenly arranged around fixation. Each search array item was positioned 260px from fixation. On each trial, the target color was selected randomly from the full set of colors, and the distractor was chosen relative to the target based on the experimental condition. In Experiments 1–3, distractor colors were equally often chosen by rotating the color wheel clockwise and counterclockwise from the target position. In Experiment 4, half of the distractors were chosen in the clockwise direction and half in the counterclockwise direction, relative to the target color. For the similarity judgment, two colored squares (80px by 80px) were presented on the left and right of the fixation point. One square was always presented in the target color, while the color of the foil square was determined by the experimental condition.

### Experiment procedure

Each trial of the task was divided into two steps (see Fig. 1A for an example trial sequence). Participants first searched for an oddball target item among 7 distractor items (6 distractors in Experiment 4). Blank placeholders were initially presented in a circular array around the fixation point for 800 ms. Then the search array items were shown in color for 200 ms, one in the target color and the remaining in the distractor color. The target item’s position was randomly determined on each trial. Participants were instructed to report the location of the briefly presented oddball target item as quickly as possible by clicking on the placeholder in the location at which they saw the target. Following their report of the target location, two colored squares were presented on either side of the fixation point, and participants were instructed to select which of the two colors was most similar to the target color from that search trial. One item was always presented in the target color, and the other color was a foil color that varied in similarity depending on experiment and condition. The similarity judgment items remained on the screen until participants made a response. We opted for a 2-AFC judgment as opposed to continuous report to minimize any possible strategic responses by participants based on their knowledge of the distractor color.

For Experiment 4, the distractors in one color (either the clockwise or counterclockwise color on the color wheel and relative to the target) were made more salient by presenting them with a thicker border (5px width compared to the 1px width of the other items). Participants were instructed that the size of the border was irrelevant to the task and to do their best to ignore it. Because of the added task difficulty, we increased visual search presentation time to 400 ms.

In Experiment 1, participants completed 300 trials of this task, with 25 repetitions of each combination of search target-distractor distance (15°, 30°, 45°, 60°, 90°, or 180°) and similarity foil color (10° rotated towards or away from the distractor color on the color wheel). In Experiment 2, participants completed a total of 432 trials, consisting of 36 trials for each combination of search target-distractor distance (30°, 60°, or 180°) and similarity foil distance (10°, 20°, 60°, or 180° rotated in the direction away from the search distractor color). In Experiment 3, participants completed a total of 288 trials, consisting of 144 visual search responses and 144 similarity judgment responses on separate trials. Visual search trials were evenly divided across the two target-distractor distances (30° or 180°), while similarity judgment trials were divided across the two target-distractor distances and similarity foil colors (foil rotated 10° towards or away from the distractor). In Experiment 4, participants completed a total of 240 trials, divided evenly across the foil color positions (10° towards or away from the salient distractor).

## Supplementary Information

Supplementary Information.

## Data Availability

Full experiment data and analysis scripts are available on OSF at https://osf.io/w9kc5/.

## References

[1] Desimone R, Duncan J (1995). Neural Mechanisms of Selective Visual Attention. Annu. Rev. Neurosci..

[2] Posner MI (1980). Orienting of attention. Q. J. Exp. Psychol..

[3] Duncan J, Humphreys GW (1989). Visual Search and Stimulus Similarity. Psychol. Rev..

[4] Carrasco M (2011). Visual attention: The past 25 years. Vis. Res..

[5] Kastner S, Pinsk MA, De Weerd P, Desimone R, Ungerleider LG (1999). Increased activity in human visual cortex during directed attention in the absence of visual stimulation. Neuron.

[6] Sprague TC, Serences JT (2013). Attention modulates spatial priority maps in the human occipital, parietal and frontal cortices. Nat. Neurosci..

[7] Williford T, Maunsell JHR (2006). Effects of spatial attention on contrast response functions in macaque area V4. J. Neurophysiol..

[8] Bisley JW, Goldberg ME (2003). Neuronal activity in the lateral intraparietal area and spatial attention. Science (80-).

[9] Luck SJ, Chelazzi L, Hillyard SA, Desimone R (1997). Neural mechanisms of spatial selective attention in areas V1, V2, and V4 of macaque visual cortex. J. Neurophysiol..

[10] Treue S, Martinez-Trujillo JC (1999). Feature-based attention influences motion processing gain in macaque visual cortex. Nature.

[11] Martinez-Trujillo JC, Treue S (2004). Feature-based attention increases the selectivity of population responses in primate visual cortex. Curr. Biol..

[12] Sàenz M (2002). Global effects of feature-based attention in human visual cortex. Nat. Neurosci..

[13] Störmer VS, Alvarez GA (2014). Feature-based attention elicits surround suppression in feature space. Curr. Biol..

[14] Carrasco M, Ling S, Read S (2004). Attention alters appearance. Nat. Neurosci..

[15] Fuller S, Carrasco M (2006). Exogenous attention and color perception: Performance and appearance of saturation and hue. Vis. Res..

[16] Liu T, Abrams J, Carrasco M (2009). Voluntary attention enhances contrast appearance. Psychol. Sci..

[17] Störmer VS, McDonald JJ, Hillyard SA (2009). Cross-modal cueing of attention alters appearance and early cortical processing of visual stimuli. Proc. Natl. Acad. Sci. USA.

[18] Störmer VS, Alvarez GA (2016). Attention alters perceived attractiveness. Psychol. Sci..

[19] Reynolds JH, Pasternak T, Desimone R (2000). Attention increases sensitivity of V4 neurons. Neuron.

[20] Treue S (2001). Neural correlates of attention in primate visual cortex. Trends Neurosci..

[21] Martínez-Trujillo JC, Treue S (2002). Attentional modulation strength in cortical area MT depends on stimulus contrast. Neuron.

[22] Navalpakkam V, Itti L (2007). Search goal tunes visual features optimally. Neuron.

[23] Scolari M, Byers A, Serences JT (2012). Optimal deployment of attentional gain during fine discriminations. J. Neurosci..

[24] Scolari M, Serences JT (2009). Adaptive allocation of attentional gain. J. Neurosci..

[25] Schurgin MW, Wixted JT, Brady TF (2020). Psychophysical scaling reveals a unified theory of visual memory strength. Nat. Hum. Behav..

[26] Chunharas C, Rademaker RL, Brady TF, Serences JT (2022). An adaptive perspective on visual working memory distortions. J. Exp. Psychol. Gen..

[27] Bae G-Y, Luck SJ (2017). Interactions between visual working memory representations. Atten. Percept. Psychophys..

[28] Scotti PS, Hong Y, Leber AB, Golomb JD (2021). Visual working memory items drift apart due to active, not passive, maintenance. J. Exp. Psychol. Gen..

[29] MacLeod DIA (2003). New dimensions in color perception. Trends Cogn. Sci..

[30] Chen J, Leber AB, Golomb JD (2019). Attentional capture alters feature perception. J. Exp. Psychol. Hum. Percept. Perform..

[31] Kriegeskorte N, Wei XX (2021). Neural tuning and representational geometry. Nat. Rev. Neurosci..

[32] Kriegeskorte N, Kievit RA (2013). Representational geometry: Integrating cognition, computation, and the brain. Trends Cogn. Sci..

[33] Carrasco M, Barbot A (2019). Spatial attention alters visual appearance. Curr. Opin. Psychol..

[34] Barbot A, Carrasco M (2018). Emotion and anxiety potentiate the way attention alters visual appearance. Sci. Rep..

[35] Tsotsos JK (1995). Modeling visual attention via selective tuning. Artif. Intell..

[36] Cutzu F, Tsotsos JK (2003). The selective tuning model of attention: psychophysical evidence for a suppressive annulus around an attended item. Vision Res..

[37] Fang MWH, Becker MW, Liu T (2019). Attention to colors induces surround suppression at category boundaries. Sci. Rep..

[38] Bartsch MV (2017). Attention to color sharpens neural population tuning via feedback processing in the human visual cortex hierarchy. J. Neurosci..

[39] Tombu M, Tsotsos JK (2008). Attending to orientation results in an inhibitory surround in orientation space. Percept. Psychophys..

[40] Yoo SA, Martinez-Trujillo JC, Treue S, Tsotsos JK, Fallah M (2022). Attention to visual motion suppresses neuronal and behavioral sensitivity in nearby feature space. BMC Biol..

[41] Moran J, Desimone R (1985). Selective attention gates visual processing in the extrastriate cortex. Science (80-).

[42] Desimone R, Wessinger M, Thomas L, Schneider W (1990). Attentional control of visual perception: Cortical and subcortical mechanisms. Cold Spring Harb. Symp. Quant. Biol..

[43] Compte A, Wang XJ (2006). Tuning curve shift by attention modulation in cortical neurons: A computational study of its mechanisms. Cereb. Cortex.

[44] Connor CE, Gallant JL, Preddie DC, Van Essen DC (1996). Responses in area V4 depend on the spatial relationship between stimulus and attention. J. Neurophysiol..

[45] Connor CE, Preddie DC, Gallant JL, Van Essen DC (1997). Spatial attention effects in macaque area V4. J. Neurosci..

[46] McAdams CJ, Maunsell JHR (1999). Effects of attention on orientation-tuning functions of single neurons in macaque cortical area V4. J. Neurosci..

[47] Suzuki S, Cavanagh P (1997). Focused attention distorts visual space: An attentional repulsion effect. J. Exp. Psychol. Hum. Percept. Perform..

[48] Bohon KS, Hermann KL, Hansen T, Conway BR (2016). Representation of perceptual color space in macaque posterior inferior temporal cortex (The V4 complex). eNeuro.

[49] Conway BR, Tsao DY (2009). Color-tuned neurons are spatially clustered according to color preference within alert macaque posterior inferior temporal cortex. Proc. Natl. Acad. Sci. USA.

[50] Brouwer GJ, Heeger DJ (2009). Decoding and reconstructing color from responses in human visual cortex. J. Neurosci..

[51] Bouchacourt F, Buschman TJ (2019). A flexible model of working memory. Neuron.

[52] Panichello MF, DePasquale B, Pillow JW, Buschman TJ (2019). Error-correcting dynamics in visual working memory. Nat. Commun..

[53] Yu X, Geng JJ (2019). The attentional template is shifted and asymmetrically sharpened by distractor context. J. Exp. Psychol. Hum. Percept. Perform..

[54] Mehrpour V, Martinez-Trujillo JC, Treue S (2020). Attention amplifies neural representations of changes in sensory input at the expense of perceptual accuracy. Nat. Commun..

[55] Magnussen S, Johnsen T (1986). Temporal aspects of spatial adaptation. A study of the tilt aftereffect. Vis. Res..

[56] Hershenson M (1989). Duration, time constant, and decay of the linear motion aftereffect as a function of inspection duration. Percept. Psychophys..

[57] Kingdom FAA (1997). Simultaneous contrast: the legaices of Hering and Helmholtz. Perception.

[58] Ekroll V, Faul F (2012). Basic characteristics of simultaneous color contrast revisited. Psychol. Sci..

[59] Becker SI, Folk CL, Remington RW (2013). Attentional capture does not depend on feature similarity, but on target-nontarget relations. Psychol. Sci..

