# Supplementary material for: Feature-based attention warps the perception of visual features
Source: Sci Rep. 2023 Apr 20;13:6487. doi: 10.1038/s41598-023-33488-2 (PMC10119379; doi:10.1038/s41598-023-33488-2)
Supplement: Supplementary file 1 — Supplementary Information. [file 41598_2023_33488_MOESM1_ESM.docx]

**Supplementary Information for**

Feature-based attention warps the perception of visual features

Angus F. Chapman, Chaipat Chunharas, and Viola S. Störmer

Corresponding author: Angus F. Chapman

Email: angusc@bu.edu

**Signal detection model of perceptual warping.** To estimate how perceptual representations of the target color were distorted by visual search, we implemented a version of the Target Confusability Competition (TCC) memory model (1), which takes a signal detection approach to estimating the representational strength of items based on the similarity between target and non-target items. TCC uses a behaviorally measured psychological similarity function to determine how a familiarity signal spreads among features based on their similarity/feature distance. Instead of measuring this similarity function, which limits the resolution to only the distances measured in the behavioral task, we constructed a version of the function reported by (1) that allowed for infinite resolution. Specifically, we assumed a population of neural tuning curves spanning the stimulus space (-180° to 180° of color space, centered on a hypothetical target at 0°). Each tuning curve was a normal distribution (SD = 11°) and they were spaced at 2° intervals across the stimulus space. To account for the circular color space, we included tuning curves out to -240° and 240°, although the final function was restricted to the actual range of the color space (-180° to 180°). We then scaled the tuning curves based on their distance from 0° by multiplying them with an exponential function: exp(-L*µ_d_/180), where µ_d_ was the absolute distance of the distribution from 0, and L scales the steepness of the exponential and was estimated from data. This roughly corresponds to the psychophysically assessed similarity function.

With this similarity function in place, we then proceeded to fit this model to the data from Experiment 2. For each target-distractor distance condition, we estimated two parameters: representational strength for the target color (measured in d’), which scales the height of the similarity function; bias (in degrees), which shifts the peak of the similarity function. We also estimated the steepness parameter L, which was shared across all target-distractor distances. Performance in the 2-AFC similarity judgment was simulated by taking the difference in the height of the similarity function between the target (0°) and foil colors, after applying the transformation by the two parameters (Figure S1). Functions were fit to the behavioral data for each subject using the “optim” function in R (version 4.0.4 (2)) with the Nelder-Mead algorithm. We then averaged the best fitting parameters for each condition across all subjects (see Table S1 for summary statistics). Final results of the fitted function are shown in Figure 2 of the main manuscript.

**Representational geometry analysis.** We assessed the changes in representational geometry as a function of attention by first simulating a hypothetical neural population. Forty simulated neurons were defined, with a firing rate modeled by a von Mises distribution (κ = 5) and a preferred feature chosen for each neuron to span the feature space (9˚ spacing; see Figure 3A in the main manuscript). Population responses were normalized by dividing by the mean neural response. To simulate the effects of attention on this neural population, we defined an “attention filter” with a gain envelope modeled by another von Mises distribution (κ = 2), centered at -20˚ to reflect a bias away from a hypothetical 30˚ distractor position. This attention filter was scaled (all values divided by the peak response), weighted (all values multiplied by 0.4), and shifted (a constant amount of 1 added to all values, resulting in increased gain for neurons selective for this feature, but no change in the response of neurons tuned at the other side of the feature space). The response of the neural population was then multiplied with this filter and normalized, giving a transformed population response under the simulated attention conditions (Figure 3C). To examine the geometry of the population, we performed multidimensional scaling (MDS) using principal components analysis (PCA) on the response properties of the simulated neural population. For these purposes, this is equivalent to using classical MDS with a Euclidian distance metric. PCA was first done on the untransformed population, and then the responses of the population modulated by attention were projected into this PC space using the component weights (Figure 3B). For plotting purposes, we used the first 2/40 PCs, which accounted for 57.1% of the variance in the simulated neural population.

**Visual search RTs as a function of target-distractor similarity.** In Experiment 1, participants reported the location of the visual search target prior to the presentation of the 2AFC similarity judgment options. RTs during visual search are generally longer the more similar the targets and distractors are (3), and we observed this in our data as well (Figure S2). Differences in RT might propagate as errors in the similarity judgment task, since biases in working memory studies have been shown to increase with delays between encoding and recall (4). To test whether this was the case in Experiment 1, we reanalyzed our data while including visual search RT as a covariate in the analysis. The results of this additional analysis are summarized in Figure S3, across a subset of simulated visual search RTs that roughly spanned the range observed in the data. These results suggest that the perceptual biases in this experiment were largely unaffected by differences in RTs from trial-to-trial.

| Target-distractor similarity | Representational strength (d’) | Bias |
| --- | --- | --- |
| 30° | 3.18 (0.50) | 11.52° (3.82) |
| 60° | 2.94 (0.42) | 5.30° (4.71) |
| 180° | 2.89 (0.44) | -3.95° (5.80) |

**Table S1.** Best fitting parameters for the signal detection model for each target-distractor similarity level. The steepness parameter L, which was shared across all similarity levels, was 7.30 (SD = 2.46).


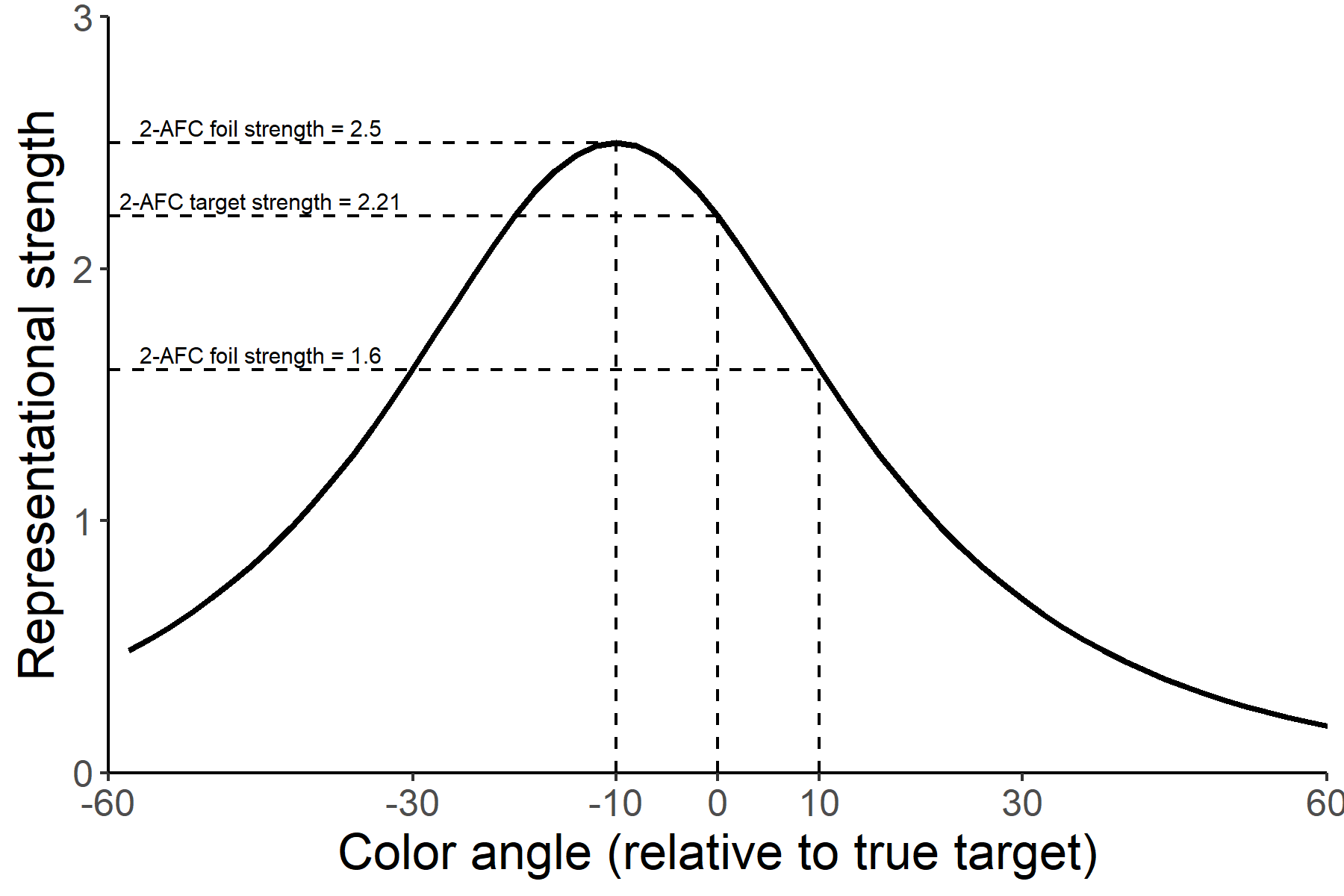


**Figure S1.** Demonstration of d’ calculation for signal detection model simulations. The representational strength varies for different colors, and in this example peaks at -10° (negative distances represent colors away from the visual search distractor). To calculate d’, we compare the representational strength of the colors in the similarity judgment: the target at 0°, and a foil at ±10°. In this example, the d’ for a 10° foil away from the distractor is 2.21-2.5 = -0.29, whereas the d’ for a 10° foil towards the distractor is 2.21-1.6 = 0.61. See main manuscript for the actual model fits.


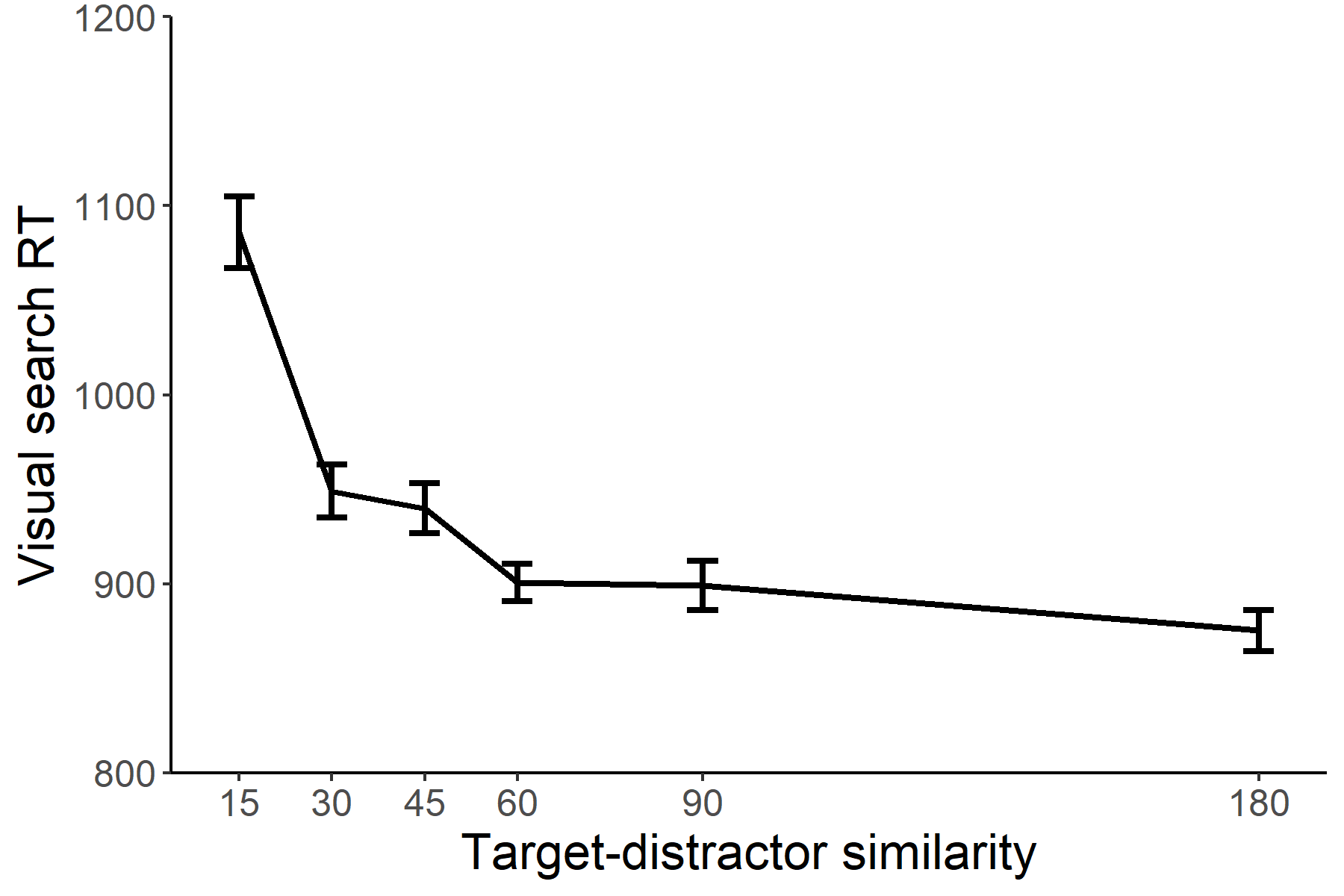


**Figure S2.** Visual search response time in Experiment 1 as a function of target-distractor similarity. In general, response times increase for less similar targets and distractors.


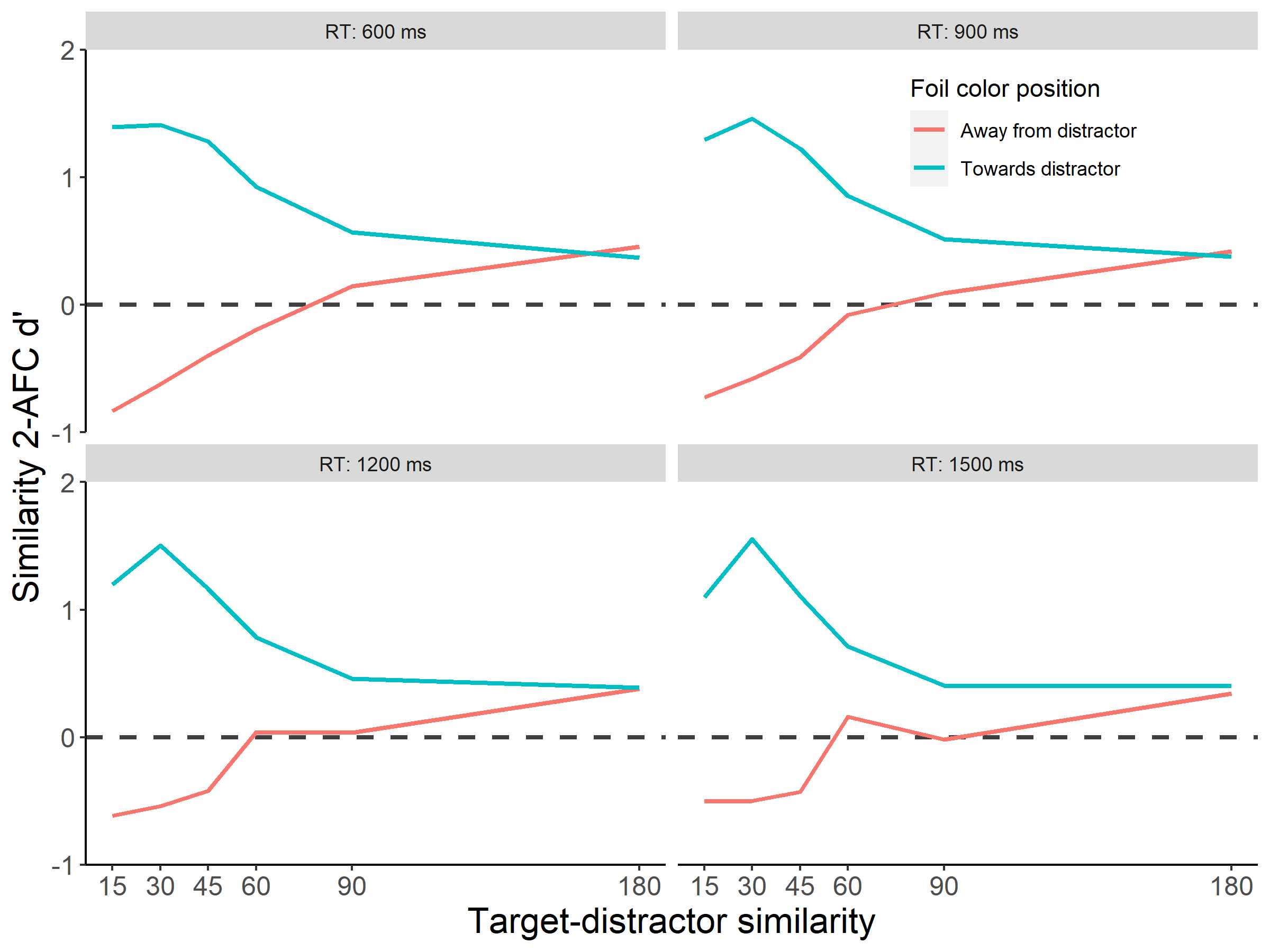


**Figure S3.** Estimated similarity judgment performance in Experiment 1 as a function of visual search RT.

Supplementary References

1. M. W. Schurgin, J. T. Wixted, T. F. Brady, Psychophysical scaling reveals a unified theory of visual memory strength. *Nat. Hum. Behav.* **4**, 1156–1172 (2020).

2. R Core Team, R: A Language and Environment for Statistical Computing (2021).

3. A. F. Chapman, V. S. Störmer, Feature similarity is non-linearly related to attentional selection: evidence from visual search and sustained attention tasks. *J. Vis.* **22**, 4 (2022).

4. C. Chunharas, R. L. Rademaker, T. F. Brady, J. T. Serences, An adaptive perspective on visual working memory distortions. *J. Exp. Psychol. Gen.* (2022) https:/doi.org/10.1037/xge0001191.
